# Optimal therapy for concomitant EGFR and TP53 mutated non-small cell lung cancer: a real-world study

**DOI:** 10.1186/s12885-023-10637-4

**Published:** 2023-03-02

**Authors:** Haiyan Sun, Peng Ren, Yongzi Chen, Lan Lan, Zhuchen Yan, Yinli Yang, Bin Wang, Cong Wang, Yanwei Li, Ling Li, Yu Zhang, Yanyang Li, Zuolin Wang, Zhanyu Pan, Zhansheng Jiang

**Affiliations:** 1 Department of Integrative Oncology, Key Laboratory of Cancer Prevention and Therapy, Tianjin Medical University Cancer Institute and Hospital, National Clinical Research Center for Cancer, Tianjin’s Clinical Research Center for Cancer, 300060 Tianjin, Tianjin, China; 2 Department of Esophageal Cancer, Key Laboratory of Cancer Prevention and Therapy, Tianjin Medical University Cancer Institute and Hospital, National Clinical Research Center for Cancer, Tianjin’s Clinical Research Center for Cancer, 300060 Tianjin, Tianjin, China; 3 Department of Tumor Cell Biology, Key Laboratory of Cancer Prevention and Therapy, Tianjin Medical University Cancer Institute and Hospital, National Clinical Research Center for Cancer, Tianjin’s Clinical Research Center for Cancer, 300060 Tianjin, Tianjin, China

**Keywords:** Epidermal growth factor receptor, Tyrosine kinase inhibitor, Combination therapy, TP53 mutations, Non-small-cell lung cancer

## Abstract

**Background:**

Non-small cell cancer (NSCLC) patients with concomitant epidermal growth factor receptor (EGFR) and TP53 mutations have a poor prognosis with the treatment of tyrosine kinase inhibitors (TKIs), and may benefit from a combination regimen preferentially. The present study aims to compare the benefits of EGFR-TKIs and its combination with antiangiogenic drugs or chemotherapy in patients with NSCLC harboring EGFR and TP53 co-mutation in a real-life setting.

**Methods:**

This retrospective analysis included 124 patients with advanced NSCLC having concomitant EGFR and TP53 mutations, who underwent next-generation sequencing prior to treatment. Patients were classified into the EGFR-TKI group and combination therapy group. The primary end point of this study was progression-free survival (PFS). The Kaplan–Meier (KM) curve was drawn to analyze PFS, and the differences between the groups were compared using the logarithmic rank test. Univariate and multivariate cox regression analysis was performed on the risk factors associated with survival.

**Results:**

The combination group included 72 patients who received the regimen of EGFR-TKIs combined with antiangiogenic drugs or chemotherapy, while the EGFR-TKI monotherapy group included 52 patients treated with TKI only. The median PFS was significantly longer in the combination group than in the EGFR-TKI group (18.0 months; 95% confidence interval [CI]: 12.1–23.9 vs. 7.0 months; 95% CI: 6.1–7.9; p < 0.001) with greater PFS benefit in TP53 exon 4 or 7 mutations subgroup. Subgroup analysis showed a similar trend. The median duration of response was significantly longer in the combination group than in the EGFR-TKI group. Patients with 19 deletions or L858R mutations both achieved a significant PFS benefit with combination therapy versus EGFR-TKI alone.

**Conclusion:**

Combination therapy had a higher efficacy than EGFR-TKI alone for patients with NSCLC having concomitant EGFR and TP53 mutations. Future prospective clinical trials are needed to determine the role of combination therapy for this patient population.

**Supplementary Information:**

The online version contains supplementary material available at 10.1186/s12885-023-10637-4.

## Introduction

Epidermal growth factor receptor (EGFR) mutations are the most common targetable oncogenic driver mutation in metastatic non-small cell lung cancer (NSCLC). EGFR tyrosine kinase inhibitors (TKIs) have shown superior efficacy over cytotoxic chemotherapy in patients with EGFR-mutant NSCLC [1–4]. However, these patients eventually experienced disease progression. The resistance is caused by the unique sub-molecular characteristics, including EGFR mutation subtypes and concurrent alterations [5]. TP53 mutations are the most prevalent co-alteration detected in 54.6–68.8% EGFR-mutant cases of NSCLC [5–9]. Patients with TP53 mutations have a low response rate and poor prognosis to EGFR-TKI therapy [10, 11].

The outcomes in patients with EGFR mutations can be improved by combining an EGFR-TKIs with an antiangiogenic agent or chemotherapy according to clinical trials suggesting the potential benefit of combination therapy [12–14]. EGFR/TP53 co-mutant tumors may preferentially benefit from a combination regimen. Both the RELAY and ACTIVE study reported that EGFR-TKIs with an antiangiogenic agent revealed consistent improved antitumor activity and favorable PFS in patients with TP53 and EGFR co-mutant NSCLC [13, 15, 16]. EGFR-TKI in combination with chemotherapy also demonstrated greater PFS benefit in patients with concurrent EGFR with TP53 mutations [17]. Nevertheless, these studies do not support subgroup analysis. Thus, this retrospective cohort study aimed to assess the efficacy of EGFR-TKIs alone or in combination with an antiangiogenic agent or chemotherapy as first-line therapy for patients with advanced NSCLC having concomitant EGFR and TP53 mutations. In current study, we analyzed and evaluated the status of TP53 and EGFR gene and the different types of mutations in relation to the outcomes of patients in terms of PFS, overall response rate (ORR), disease control rate (DCR), and duration of response.

## Materials and methods

### Patient eligibility

We retrospectively reviewed data from patients with local advanced stage and metastatic NSCLC having concomitant EGFR and TP53 activating mutations admitted in the Tianjin Medical University Cancer Institute and Hospital(Tianjin, China) between January 2018 and June 2021. The patients who met the following inclusion criteria were included: (1) pathological confirmed, (2) harboring concomitant EGFR and TP53 mutations, as confirmed by next-generation sequencing (NGS), (3) receiving EGFR-TKIs treatment, and (4) regularly imaged for the effective analysis based on the Response Evaluation Criteria in Solid Tumors (RECIST, version 1.1). Patients who met the following criteria were excluded: (1) concurrently or previously diagnosed with other malignancies, (2) with insertional mutations in exon 20, and (3) with a follow-up period of no more than 6 months. Approval for a waiver of informed consent for the study was obtained from the Institutional Review Board of the Tianjin Medical University Cancer Institute and Hospital. All methods were performed in accordance with the relevant guidelines and regulations. Informed consent was not required as this is a retrospective, unicentric cohort study.

### Study endpoints and assessment

PFS was defined as the primary endpoint, which represents the time between treatment initiation and the last follow-up or cancer progression. In addition, ORR, DCR, and duration of response (DoR) were deemed as secondary endpoints. We evaluated ORR through the partial response (PR) and complete response (CR) rates, while DCR was evaluated based on PR, CR, and stable disease (SD) rates. DoR was defined as the time from the first documented response to the onset of disease progression or death, whichever occurred first.

### Treatment method

Patients were classified into the EGFR-TKI group and combination therapy group. The patients in EGFR-TKI group received first-, second-, or third- generation EGFR-TKI drugs only. The EGFR-TKI drugs include gefitinib, icotinib, erlotinib, afatinib, dacomitinib, osimertinib, and almonertinib. The patients in the combination group underwent EGFR-TKIs combined with anti-angiogenic drugs or chemotherapy. The antiangiogenic drugs include bevacizumab and anlotinib. The chemotherapy regimens include pemetrexed and carboplatin or cisplatin. There was no direct involvement of human tissues.

### Data collection

Patient characteristics including gender, age, smoking history, Eastern Cooperative Oncology Group (ECOG) score, disease status, number of organs with metastases, metastatic sites, EGFR and TP53 mutations status, treatment status, and concomitant mutations other than EGFR and TP53 were obtained from the electronic medical record system of the Tianjin Medical University Cancer Hospital and Institute. The last follow-up was conducted on January 20, 2022. The efficacy was evaluated using the Response Evaluation Criteria in Solid Tumors Version 1.1 (RECIST 1.1). Data were considered as censored if the event had not occurred by the last follow-up time.

### Next-generation target sequencing

The tissue DNA was extracted using the QIAamp DNA FFPE Tissue Kit (Qiagen, Valencia, CA, USA) according to the manufacturer’s instructions. Circulating cfDNA was recovered from 4 to 5 mL plasma by using the QIAamp Circulating Nucleic Acid kit (Qiagen). The tissue DNA and cfDNA were profiled using a capture-based targeted sequencing panel that consisted of 520 or 168 cancer-related genes. The captured libraries were sequenced on Illumina NovaSeq 6000 with pair-end reads with 1000X for tissue DNA and 20,000 X for cfDNA, following the manufacturer instructions (Illumina, San Diego, CA, USA).

### Statistical analysis

The last follow-up in this study was conducted in January 2022. Subgroup analysis was conducted based on age, gender, smoking status, ECOG score, clinical stage, bone metastases, brain metastases, TP53 mutation types, EGFR mutation types, and other concomitant gene mutations. Differences between groups were compared using chi-square test. Kaplan–Meier (KM) curves were plotted to analyze PFS, and log-rank test was used to compare differences between groups. Univariate cox regression analysis was performed on the risk factors associated with survival. Fisher’s exact test or chi-square test was used to analyze DCR and ORR. Hazard ratios (HRs) and 95% confidence intervals (CIs) were estimated using stratified Cox regression. The one-year PFS rate and the corresponding 95% CIs for each treatment group were calculated using the Greenwood formula. SPSS 26.0 (IBM Corp.) was employed for statistical analysis. Two-sided p-values < 0.05 were considered statistically significant.

## Results

### Clinical characteristics of patients

**Fig. 1:**
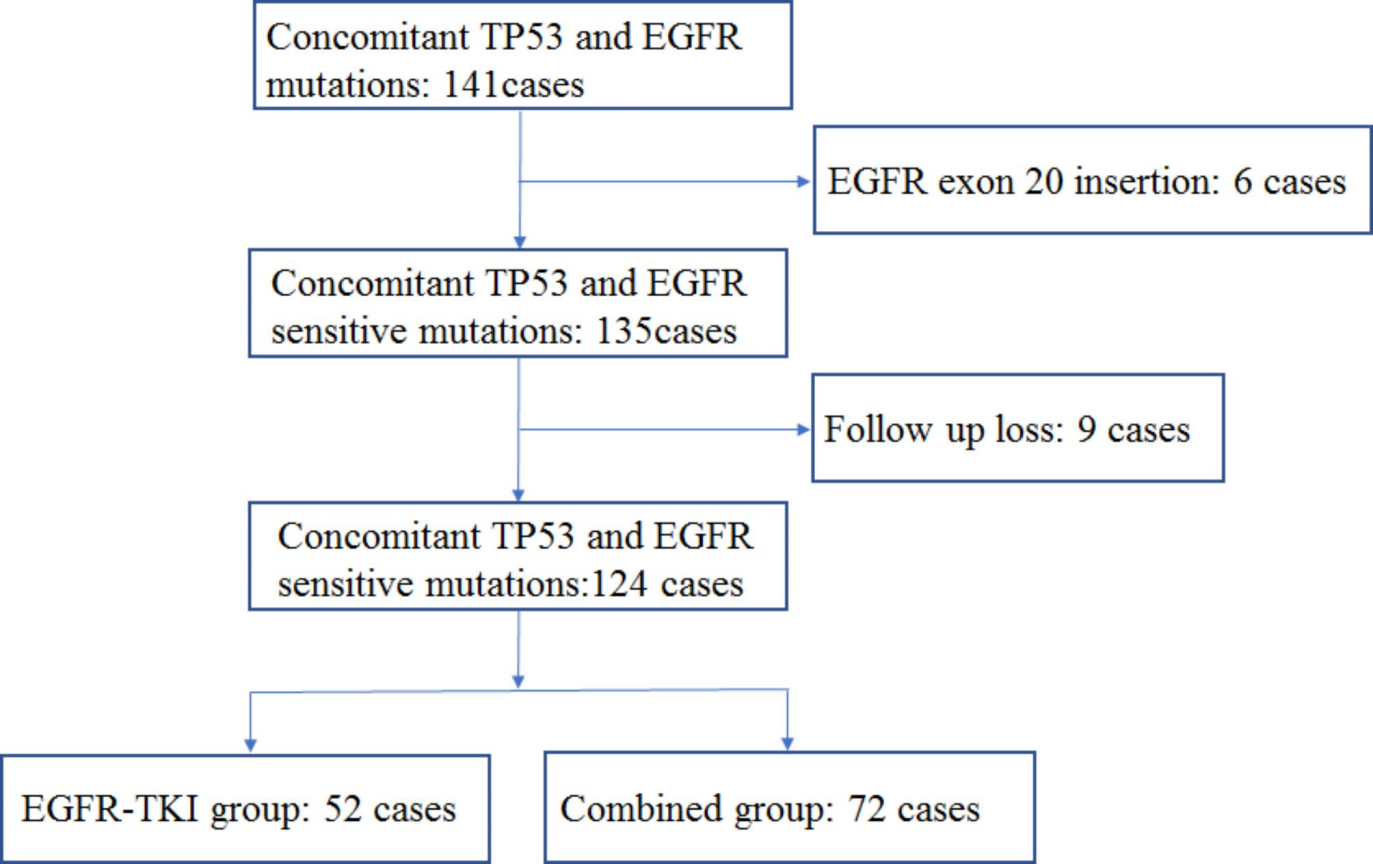
The flow diagram representing patient enrollment in the study

Among the 141 potentially eligible patients, we excluded six patients with insertional mutations in exon 20. Additionally, we excluded nine patients with missing follow-up information. Therefore, 124 NSCLC cases harboring EGFR and TP53 mutations were included in this study (Fig. 1). A total of 52 patients underwent EGFR-TKI treatment (EGFR-TKI group), including 31 with first-generation EGFR-TKIs, 5 with second-generation EGFR-TKIs, and 16 with third-generation EGFR-TKIs, respectively. A total of 72 patients underwent EGFR-TKIs combined with antiangiogenic drugs (22 cases) or chemotherapy (50 cases). In the EGFR-TKIs combined with antiangiogenic drugs subgroup, 12 cases were treated with anlotinib and EGFR-TKIs, and 10 with bevacizumab and EGFR-TKIs. The median chemotherapy cycle was 6 (2–34). A similar proportion of patients were present in both groups based on gender, age, smoking history, ECOG score, clinical stage, number of organs with metastases, EGFR mutations, TP53 mutations and treatment status, and concomitant mutations, except for the combination group with more patients having bone metastases than the EGFR-TKI group (p = 0.043). Twelve (23.1%) patients in the EGFR-TKIs group and 20 (27.8%) in the combination group had coexisting brain metastases, while 23 (44.2%) patients in the EGFR-TKIs group and 46 (62.5%) in combination group had coexisting bone metastases (Table 1).

**Table 1:** Clinicopathological characteristics of patients

Variable	EGFR-TKI(n = 52)	Combination therapy(n = 72)	P value
Age, years			0.319
Median	58(36–84)	58(33–76)	
< 65	38(73.1%)	56(77.8%)	
≥ 65	14(26.9%)	16(22.2%)	
Sex, n (%)			0.198
Male	18(34.6%)	34(47.2%)	
Female	34(65.4%)	38(52.8%)	
Smoking habits, n (%)			0.846
Never smoker	37(71.2%)	50(69.4%)	
Ever smoker	15(28.8%)	22(20.6%)	
ECOG performance status			0.535
0	15(28.8%)	26(36.1%)	
1	35(67.3%)	45(62.5%)	
2	2(3.9%)	1(1.4%)	
Disease status			0.148
Advanced	8(15.4%)	5(6.9%)	
Metastases	44(84.6%)	67(93.1%)	
Number of organs with metastases			0.538
≤ 1	15(28.8%)	17(23.6%)	
≥ 2	37(71.2%)	55(76.4%)	
Site of metastases			
Lymph node	38(73.1%)	50(69.4%)	0.693
Pleura	18(34.6%)	23(31.9%)	0.847
Liver	6(11.5%)	9(12.5%)	0.871
Bone	23(44.2%)	46(63.9%)	0.043
Brain	12(23.1%)	20(27.8%)	
EGFR mutation type, n (%)			0.361
Exon 19 del	27(51.9%)	29(40.3%)	
p.L858R	20(38.5%)	36(50%)	
Other	5(9.4%)	7(9.7%)	
Concomitant other mutations			0.842
Yes	38(73.1%)	51(70.8%)	
No	14(26.9%)	21(19.2%)	
TP53 alterations			0.460
Missense variant	33(63.4%)	40(55.6%)	
Nonsense	9(17.3%)	12(16.7%)	
Frameshift	3(5.8%)	8(11.1%)	
Splice site	3(5.8%)	8(11.1%)	
Indel	0	3(4.2%)	
Inframe insertion	1(1.9%)	0	
Unknown	4(7.7%)	2(2.8%)	
TP53 exon			
Exon 8	7(13.5%)	15(20.8%)	0.470
Non exon 8	45(86.5%)	58(79.2%)	
Exon 4/7	11(21.2%)	22(30.6%)	0.529
Non exon 4/7	41(78.8%)	52(69.4%)	

ECOG, Eastern Cooperative Oncology Group, EGFR, epidermal growth factor receptor

### Baseline genomic characteristics

#### EGFR mutations

Among the 124 patients with baseline tissue or plasma samples detected with gene mutations by NGS, 56 (45.2%) exon 19 deletions (19 del), 56 (45.2%) exon 21 L858R, 2 L861Q, 1 L814P, 1 E709_T710delinsD, and 7 (4.0%) compound EGFR mutations (including 2 with G719S and S768I, 1 with G719D and L861Q, 1 with G719S and E709K, 1 with 19 deletion and L858R, 1 with E709A and L858R, 1 with E709K and L858R, and 1 with 19 deletion and P848L) were observed.

#### TP53 mutations

**Fig. 2:**
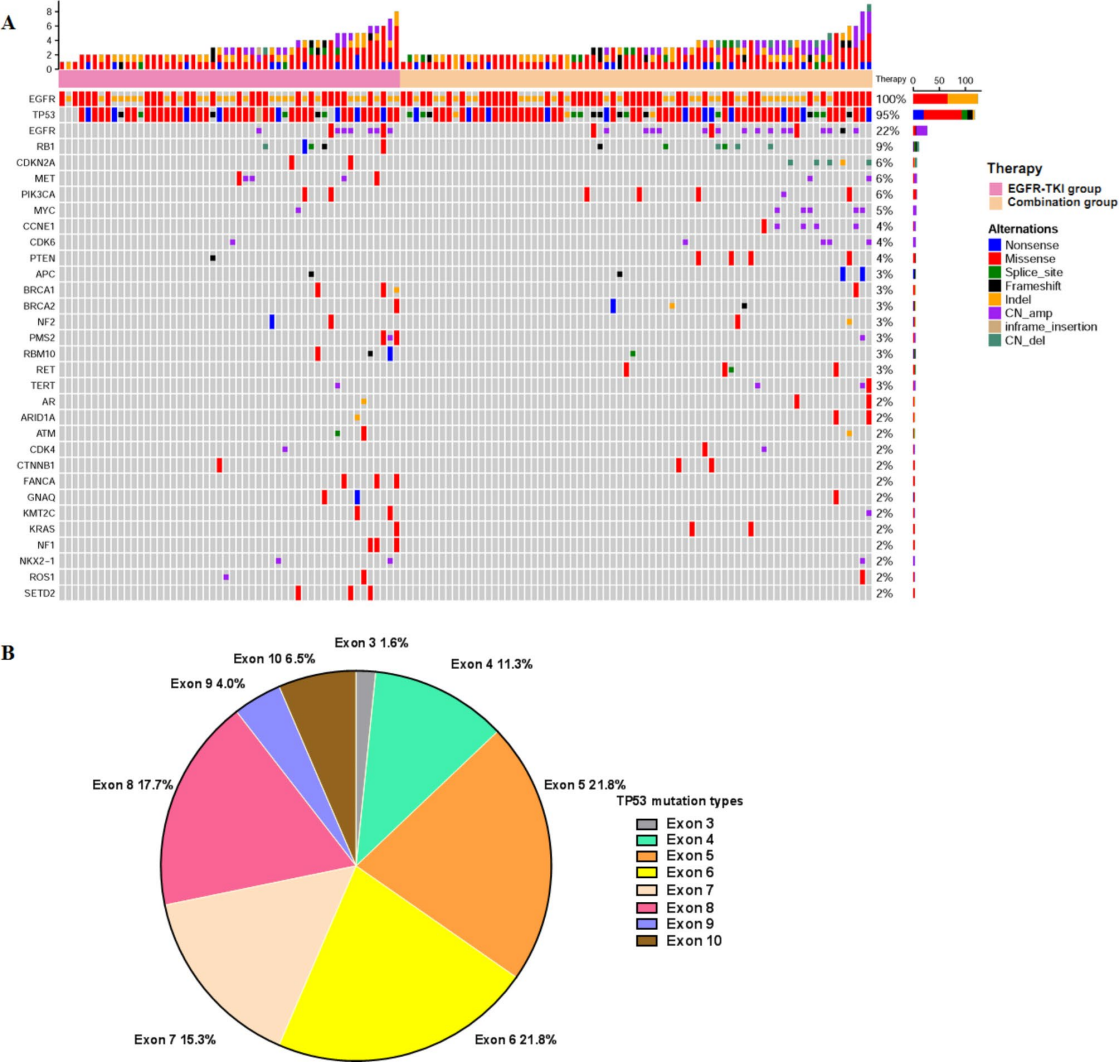
Gene Landscapes and Details of TP53. (A) Oncoprint of genomic alterations identified in baseline tumor tissue and/or plasma samples (n = 124); (B) TP53, mutations detected in patients at baseline

Among the 124 EGFR-mutated patients, 118 cases had detailed information on TP53 mutation analysis. The TP53 mutations in 6 patients were only based on medical records without detailed information. Figure 2B shows the percentages of all exon mutations of TP53 detected in patients at baseline. The most frequent mutation in TP53 exons occurred in exon 5 and 6 (both 21.8%, *n* = 27), followed by exon 8 (17.7%, *n* = 22) and exon 7 mutation (15.3%, *n* = 19). Fourteen (13.1%) patients harbored exon 4 mutations. Eight (6.5%) patients harbored exon 10 mutations. Five (4.0%) patients exhibited TP53 mutation on exon 9, 2 (1.6%) on exon 3. TP53 exon 1, 2, and 11 mutations were not found in this cohort.

No statistically significant associations were observed between the type of TP53 and EGFR mutation.

#### Other concomitant mutations

Twenty-one patients (16.9%) harbored co-occurring lung cancer driver alterations, including one ERBB2 amplification (amp), one ERBB2 mutation, four RET mutations, five MET amp, one ROS1 amp, two MET mutations, two ALK mutations, one BRAF V600E, one BRAF mutation and one KRAS G13C, one KRAS G13D and one KRAS G12V. Among the common concomitant alterations included other EGFR alternation (17.7%, 22/124), RB1 (8.9%, 11/124), CDKN2A (5.6%, 7/124), APC (3.2%, 4/124), MYC (4.8%, 6/124), PIK3CA (5.6%, 7/124), CTNNB1 (2.4%, 3/124), TERT (3.2%, 4/124), NF2 (3.2%, 4/124), PTEN (4%, 5/124), BRAC1 (5%, 5/124), and CDK6 (5%, 5/124), as shown in Fig. 2A.

### Efficacy

#### Progression-free survival

**Fig. 3:**
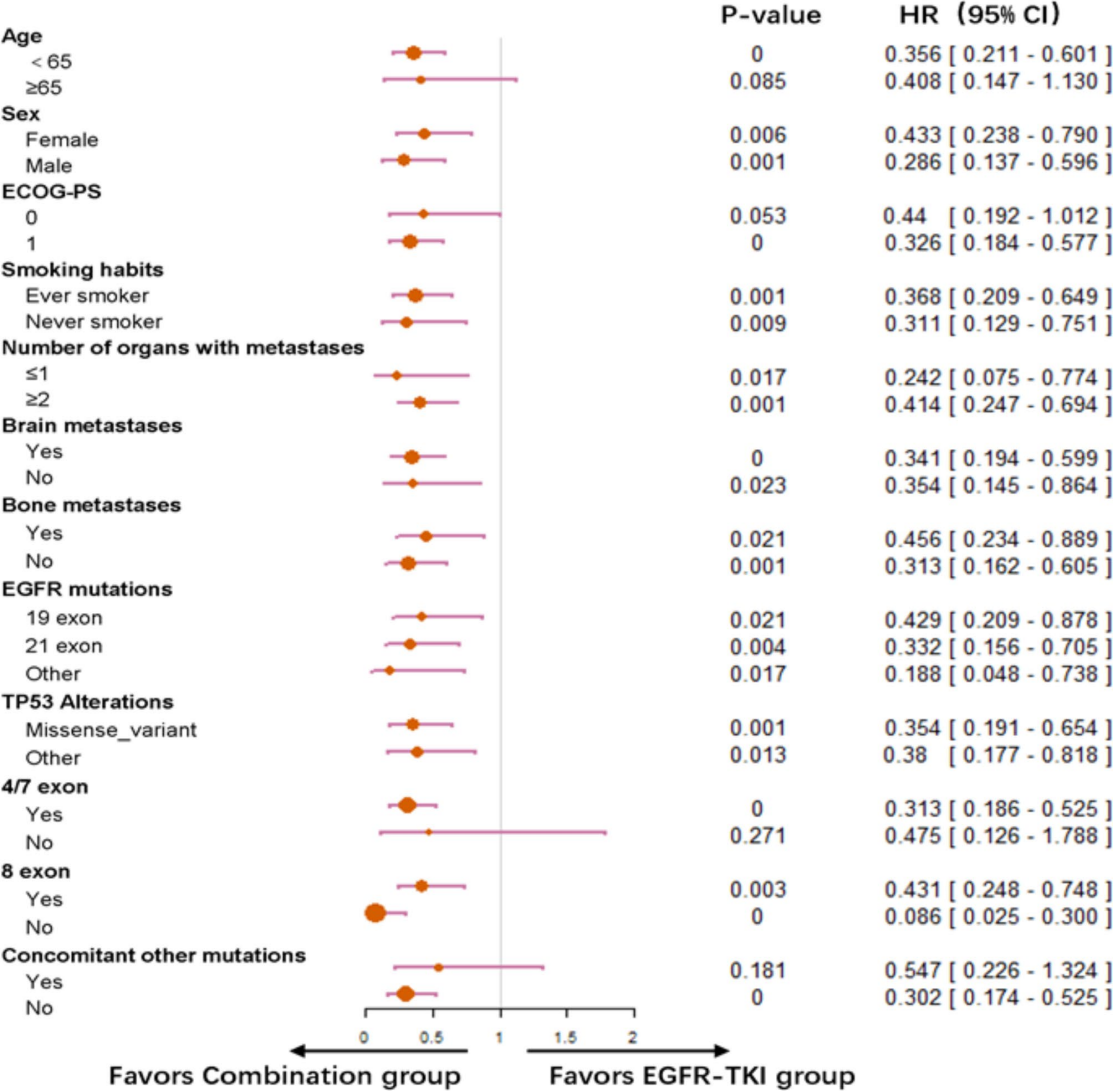
Kaplan-Meier curves of progression-free survival (PFS) of patients with different therapies. (A) Patients with EGFR-TKI only and combination therapy. (B) Patients with 1st and 2nd generation EGFR-TKI, 3rd generation EGFR-TKI and combination therapy. (C) Patients with 1st ,2nd and 3rd generation EGFR-TKI in EGFR-TKI group. (D) Patients with EGFR-TKI combined with antiangiogenic drugs and EGFR-TKI combined with chemotherapy

Progression events occurred in 40 (76.9%) patients in the EGFR-TKI group and in 37 (51.4%) patients in the combination group. The duration of PFS was significantly longer in the combination group than in the EGFR-TKI group (median, 18.0 months [12.1–23.9] vs. 7.0 months [6.1–7.9]; HR: 0.37; 95% CI: 0.232–0.589; p < 0.001, Fig. 3A). The estimated proportion of patients who were alive and progression-free at 6 months was 89% (95% CI: 81–95) in the combination group and 71% (95% CI: 59–84) in the EGFR-TKI group. At 12 months, the proportions were 62% (95% CI: 49–74) and 24% (95% CI: 22–25). At the time of data cutoff, 77 progression events occurred (62% maturity), representing the planned number of events and maturity. The follow-up lasted for 6–30 months with the median time of 12 months.

**Fig. 4:**
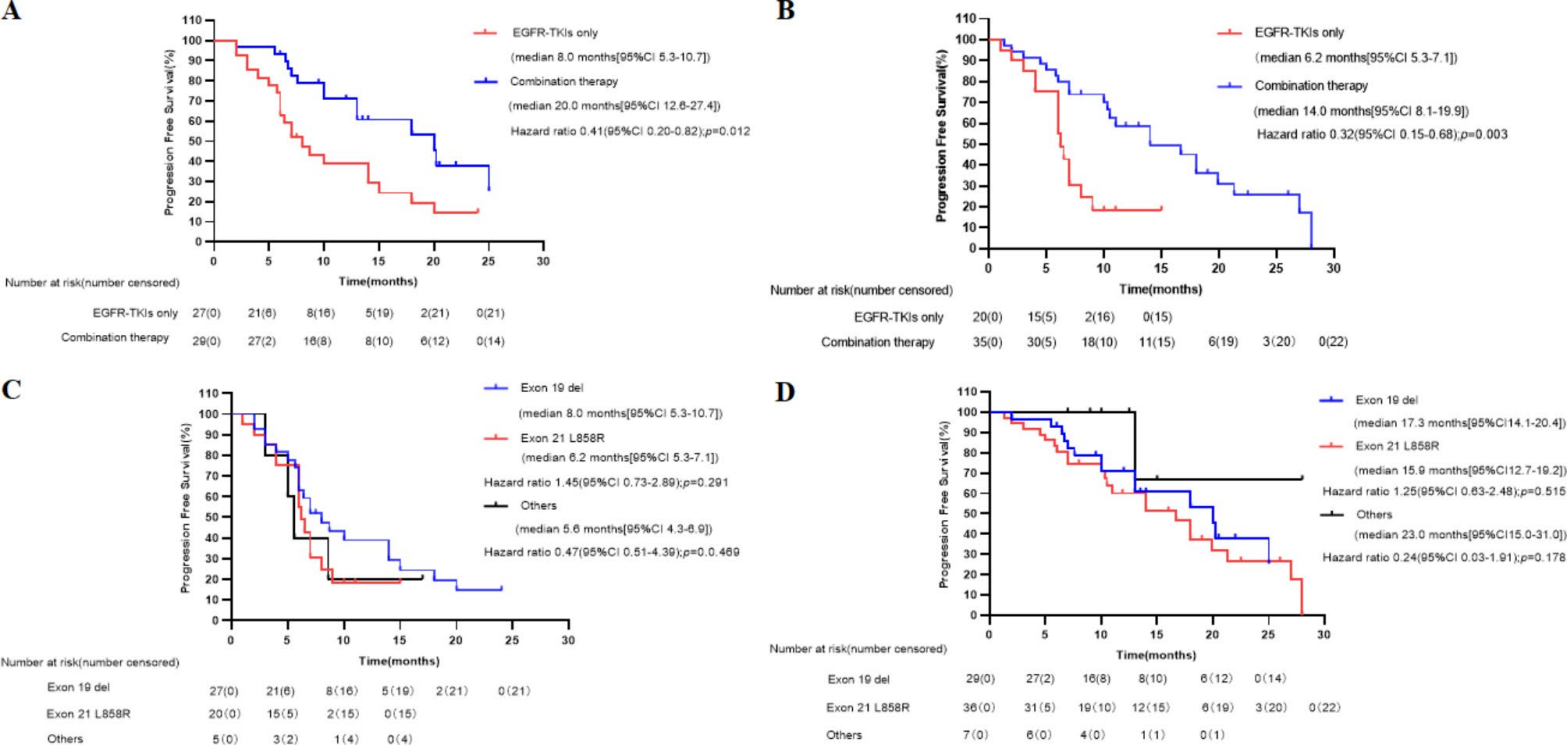
Forest plot of subgroup analysis of progression-free survival. ECOG-PS, Eastern Cooperative Oncology Group performance status

Varying PFS benefit from combined therapy was observed across all predefined subgroups compared with the EGFR-TKI group (Fig. 4). Treatment option was the only independent factor for PFS in the univariate and multivariate Cox models (Supplementary Table 1).

In the EGFR-TKI group, the PFS of patients treated with first-, second-, and third-generation EGFR-TKIs (30, 7, 15 patients respectively) had no difference (median, 6.2 months [5.6–6.8] months vs. 9.0 months [8.0–10] months vs. 14.0 months [2.2–25.8] months; p = 0.295), as shown in Fig. 3C. Third-generation EGFR-TKIs had no superiority over other TKIs when first- and second-generation EGFR-TKIs were combined into one group (median, 14.0 months [2.2–25.8] months vs. 6.4 months [5.7–7.0] months; p = 0.193), as shown in Fig. 3B. In the combination group, we compared the PFS of patients treated with EGFR-TKIs combined with antiangiogenic drugs or EGFR-TKIs combined with chemotherapy and found no difference between two subgroups (median, 18 months [14.1–22.0] vs. 19.9 months [12.5–27.3]; HR: 0.88; 95% CI: 0.446–1.775; p = 0.122), as shown in Fig. 3D.

#### Responses in different EGFR subtypes

**Fig. 5:**
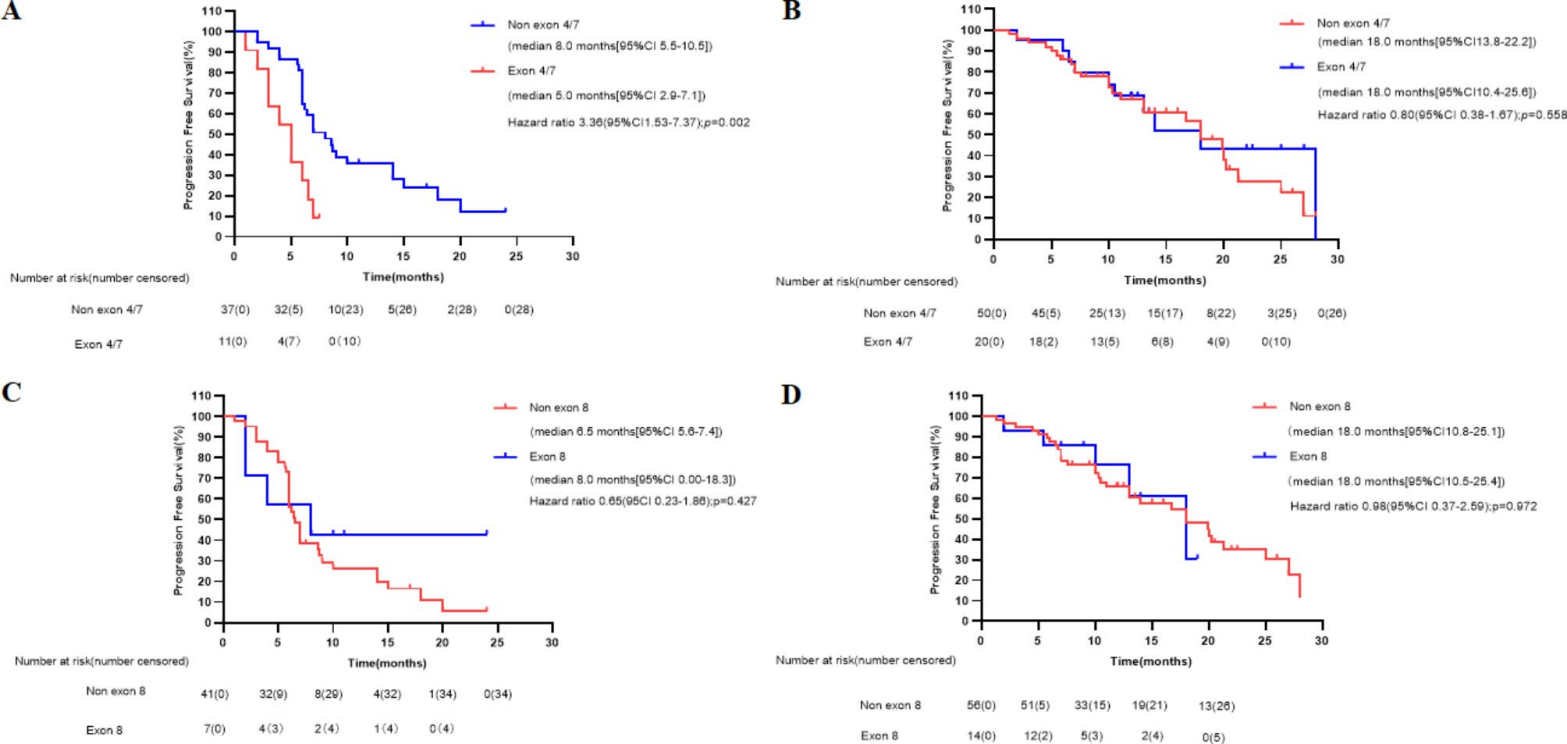
Kaplan-Meier curves of progression-free survival (PFS) of patients according to EGFR mutations. (A) Patients with EGFR exon 19 del. (B) Patients with exon 21 exon L858R mutation. (C) Patients with 19 del, L858R and other mutations in EGFR-TKI group. (D) Patients with 19 del, L858R and other mutations in combination therapy group

Regarding the association between the type of EGFR mutation and therapy response, patients with 19 deletion or L858R mutations both achieved a significant PFS benefit after combination therapy compared with EGFR-TKIs alone, and PFS was similar between 19 deletions, L858R, and other mutations (Fig. 5).

#### Analysis among TP53 subtypes

**Fig. 6:**
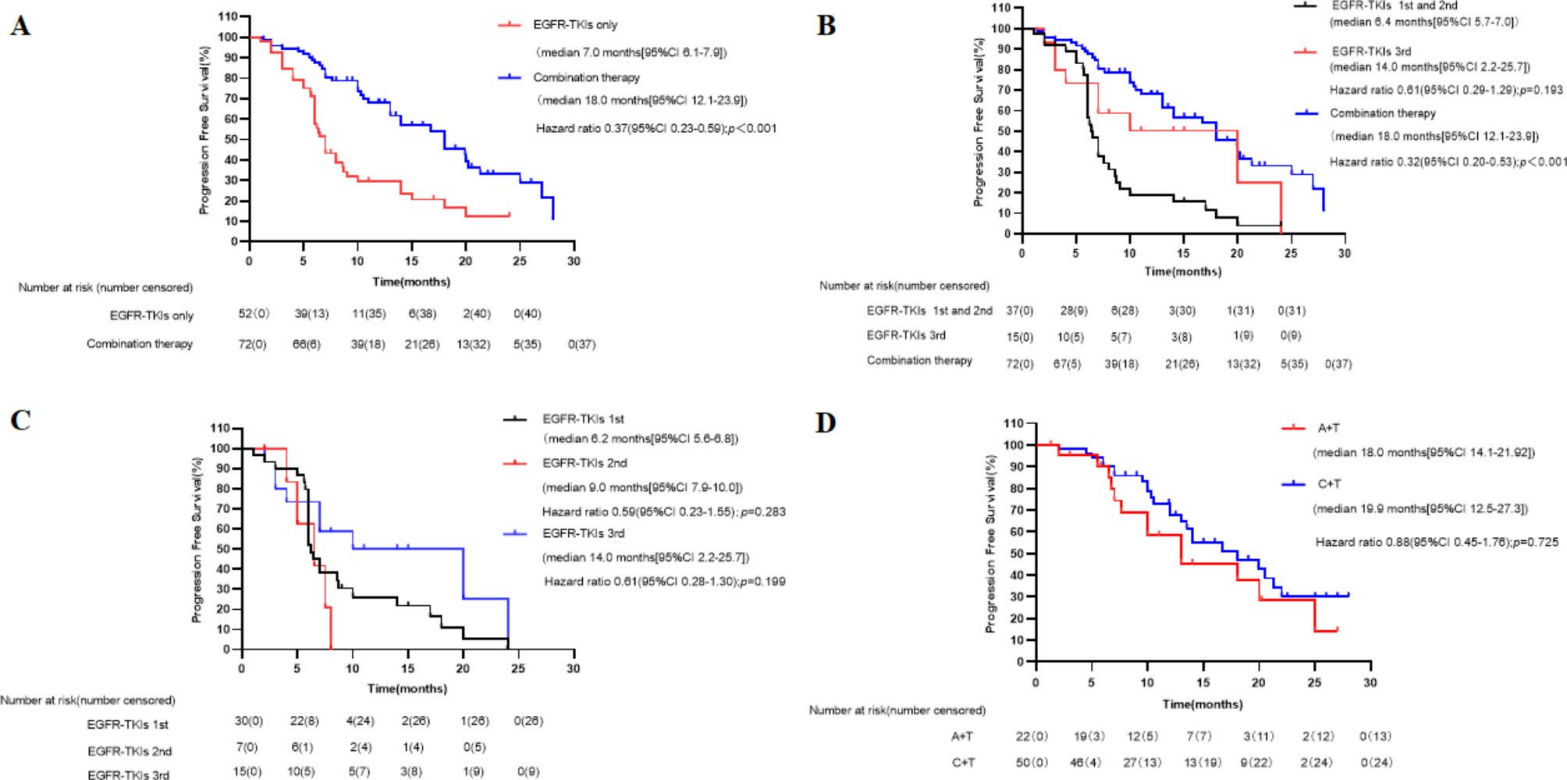
Kaplan-Meier curves of progression-free survival (PFS) of patients according to TP53 mutations. (A) Patients with TP53 exon 4/7 vs. non exon 4/7 in EGFR-TKI group. (B) Patients with TP53 exon 4/7 vs. non exon 4/7 in combination therapy group. (C) Patients with TP53 exon 8 vs. non exon 8 in EGFR-TKI group. (D) Patients with TP53 exon 8 vs. non exon 8 in combination therapy group

Considering the different TP53 mutations, patients with exon 4 or 7 (4/7) mutations had shorter PFS than patients harboring non-exon 4/7 mutations in EGFR-TKI group (Fig. 6A). However, in the combination therapy group, the patients achieved a similar PFS benefit regardless of exon 4/7 mutations in TP53 (Fig. 6B). A greater PFS benefit was observed in TP53 exon 4/7 mutations subgroups. No significant difference was observed in patients stratified by other TP53 mutation type (Fig. 6C and D),

#### Genomic predictors of PFS outcome

**Fig. 7:**
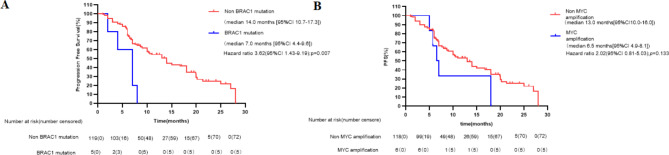
Kaplan-Meier curves of progression-free survival (PFS) of patients according to other concomitant mutations. (A) Patients with BRAC1 mutation vs. non BRAC1 mutation. (B) Patients with MYC amplification vs. non MYC amplification

BRAC1 mutation had a significantly shorter PFS than wild-type BRAC1 (median, 7.0 [95% CI: 4.4–9.6] months vs. 14.0 [95% CI: 10.7–17.3] months; p = 0.007; Fig. 7A). MYC amplification tend to be associated with shorter PFS (median, 6.5 [95% CI: 4.9–8.1] months vs. 13 [95% CI: 10.0–16.0] months; p = 0.133), especially in the combination therapy group (median, 7.0 [95% CI: 5.9–8.1] months vs. 18.0 [95% CI: 14.3–21.7] months; p = 0.036; Fig. 7B).

#### ORR, DOR, and DoR

In the EGFR-TKI group, 23 (44.2%) of patients showed a partial response (PR), and 23 (44.2%) had SD; in the combination group, 40 (55.6%) had PR, and 29 (40.3%) had SD. The patients in two groups had comparable ORRs [44.2% [95% CI: 30.3–58.2%] vs. 55.6% [95% CI: 43.8–67.3%; OR, 0.97; p = 0.275]). Furthermore, 46 (88.5%, 95% CI: 79.5–97.6%) patients in the EGFR-TKIs and 69 (95.8%, 95% CI: 91.1–100%) patients in the combination group achieved disease control (p = 0.164). The median duration of response was significantly longer in the combination group than in the EGFR-TKI group (median, 13.2 [95% CI:10.4–16.2] months vs. 9.0 [95% CI: 7.0–11.1] months; p = 0.039) (Table 2).

**Table 2:** Summary of responses in the EGFR/TP53 co-mutation population

End Points	EGFR-TKI(N = 52)	Combined therapy(N = 72)	P value
Best objective response			
Complete response	0	0	
Partial response	23(44.2)	40(55.6)	
Stable disease	23(44.2)	29(40.3)	
Progressive disease	6(11.6)	3(4.1)	
Objective response	23(44.2)	40(55.6)	0.275
Disease control	46(88.5)	69(95.8)	0.164
Time to progressive disease			
Events	40(76.9)	37(51.0)	
Median (95% CI)	6.9(5.7 to 8.2)	11.4(9.3 to 14.3)	0.001
Hazard ratio		0.427	
Duration of response			
Events, n/N responders (%)	15/23(65)	20/40(50)	
Median (95% CI)	9.0(7.0 to 11.1)	13.2(10.4 to 16.2)	0.039
Hazard ratio		0.476	

## Discussion

In the present study, we selected patients with EGFR and TP53 co-mutation and retrospectively analyzed the PFS in patients with different treatment options. To the best of our knowledge, this study was the first to analyze optimal treatment options for EGFR and TP53 co-mutant NSCLC patients in a relatively large case series. We found that for patients with concomitant concurrence of EGFR and TP53 mutations, the duration of PFS was significantly longer with EGFR-TKI treatment combined with antiangiogenic drugs or chemotherapy than with EGFR-TKIs only, regardless of whether first-, second-, or third-generation EGFR-TKI was used. These data were even more significant for patients with TP53 exon 4 or 7 mutations.

The p53 protein regulates cellular responses to various cellular stress signals by inducing cell-cycle arrest, senescence, or apoptosis [18]. The complete loss of TP53 function can accelerate the transformation potential of driver oncogenes in lung cancers [19]. TP53 mutations play a role in predicting poor prognosis of patients with advanced NSCLC [20, 21]. TP53 mutations is an unfavorable prognostic factor in patients receiving first-, second-, and third-generation EGFR-TKI therapy with EGFR mutant advanced NSCLC [7, 11, 22, 23]. TP53 mutation status can be used to select treatment for patients with EGFR mutated lung cancer. In the present study, we also compared the PFS of patients treated with first-, second-, and third-generation EGFR-TKIs and found comparable PFS among the treatments with lower PFS compared with the efficacy of EGFR TKIs reported in the literature.

The role of TP53 in angiogenesis has also been established, and multiple studies have shown that the presence of TP53 mutation is associated with the upregulation of the VEGF pathway and may predict clinical sensitivity to antiangiogenic therapies in several tumor types [24, 25]. Clinical studies have also shown that for patients with concomitant TP53 mutation, the combination regimen of EGFR-TKIs and antiangiogenic drugs improved the PFS compared with TKI only [16, 26]. The combination of chemotherapy and EGFR-TKIs also eliminated this heterogeneity of TP53 co-mutation [17]. In our study, PFS was significantly longer with EGFR-TKI treatment combined with antiangiogenic drugs or chemotherapy than with EGFR-TKIs only. We also compared the PFS of patients treated with antiangiogenic drugs or chemotherapy and found that the efficacy of the two regimens is comparable.

EGFR mutation types exhibit different biology after treatment with EGFR-TKIs therapy, with improved outcomes in patients harboring exon 19 deletions compared with L858R [27–29]. For patients with EGFR/TP53 co-mutation, higher ORR and PFS was observed in the subgroup of patients with exon 19 deletion with respect to patients with L585R mutation [16, 23]. In the present study, patients with 19 deletion or L858R mutations both achieved a significant PFS benefit after combination therapy compared with EGFR-TKI alone, but no difference was observed between 19 deletion and L858R. The survival advantage of 19 deletion may be neutralized by the negative effects of TP53 mutations.

Classifying TP53 mutations has become increasingly important, because mutants in different exons exhibit different biological effects and clinical implications [9, 11, 16, 23]. Potential optimization strategies for certain subgroups of patients with baseline EGFR/TP53 co-mutated status must be explored. Mutations in the exon 4 or 7 of TP53 in patients with NSCLC were correlated with worse PFS than in patients with other exons [9]. We compared the PFS of patients with 4 or 7 of TP53 mutations and other TP53 mutations and found PFS benefits tended to favor the combination group in the subgroup of exon 4 or 7 mutations. Canale and Zhao H [11, 16, 23] reported a shorter median PFS in patients with EGFR mutant NSCLC with TP53 exon 8 mutations compared with other exon subsets. In the present study, patients with exon 8 of TP53 mutations achieved a similar PFS benefit in the EGFR-TKIs group and the combination group, and had similar median PFS to the overall patient population with the same therapy group.

Co-occurring genomic alterations such as RB1 mutation, PTEN mutation, and MDM2 and CDK4/6 amplification are associated with worse PFS in patients with EGFR mutation-positive NSCLC [30, 31]. However, in our EGFR and TP53 co-mutation cohort, these alternations were not associated with PFS. BRAC1 mutation and MYC amplification predicted poor prognosis for EGFR-TKI therapy or combination therapy. The amplification and overexpression of MYC were found in approximately 10% NSCLC [32]. MYC amplification was correlated with chemotherapy resistance in lung cancers [33]. The enforced expression of ectopic MYC partially protects sensitive EGFR mutant cells from undergoing osimertinib-induced apoptosis and decreases cell survival [34]. APC alternations are also associated with shorter PFS in patients treated with gefitinib [26], which was consistent with our study. Low BRAC1 expression levels were associated with increased PFS after platinum-based chemotherapy [35]. Our study was the first to report that BRAC1 was associated with unfavorable PFS after EGFR-TKI treatment or combination therapy. Nevertheless, considering the small number of BRAC1 and MYC mutated patients, the study was not powered for subgroup analysis. Thus, the results of the analysis of BRAC1 and MYC alternations should be interpreted with caution.

The present study was limited by its retrospective nature, the use of a single accrual center, and lack of data validation from external multicenter, which restricted our ability to investigate other sources of potential bias. Considering that we included patients that received different first-, second-, third-generation EGFR-TKIs treatment and different combination regimens, and the TP53 mutations in six patients were only recorded based on medical records without detailed information, the generalizability of our study findings must be considered. In addition, this study did not include the analysis of adverse effects because of the incomplete medical records. Therefore, further prospective studies conducted in larger cohorts are required to validate the efficacy of combination therapy observed in the study.

## Conclusion

This study was the first to present survival outcomes to compare the benefits of EGFR-TKIs and its combination with antiangiogenic drugs or chemotherapy in EGFR and TP53 co-mutation patients. In comparison with EGFR-TKI monotherapy, EGFR-TKIs combined with antiangiogenic drugs or chemotherapy significantly improved PFS in patients with advanced NSCLC having concomitant EGFR and TP53 mutations.

## Electronic supplementary material

Below is the link to the electronic supplementary material.

Supplementary Material 1

## Data Availability

The datasets during the current study are not publically available due to patient confidentiality but can be obtained from Haiyan Sun upon sufficient and reasonable request.
