# Supplementary material for: Optimal therapy for concomitant EGFR and TP53 mutated non-small cell lung cancer: a real-world study
Source: BMC Cancer. 2023 Mar 2;23:198. doi: 10.1186/s12885-023-10637-4 (PMC9979422; doi:10.1186/s12885-023-10637-4)
Supplement: Supplementary file 1 — Supplementary Material 1 [file 12885_2023_10637_MOESM1_ESM.docx]

Supplementary Table 1 Univariate and multivariate analyses of predictive factors for PFS

|  |  | Univariate analysis | Maltivariant analysis |
| --- | --- | --- | --- |
| Variables |  | P value | P value |
| Therapy |  | <0.001 | <0.001 |
| Age | ＜65 vs ≥65 | 0.454 | 0.525 |
| Sex | Female vs male | 0.196 | 0.903 |
| Eastern Cooperative Oncology Group performance status | 0 vs 1 vs 2 | 0.818 | 0.477 |
| Smoking status | Ever vs never | 0.457 | 0.423 |
| Disease status | Stage Ⅳ vs others | 0.893 | 0.835 |
| Brain metastases | Yes vs no | 0.459 | 0.424 |
| Bone metastases | Yes vs no | 0.091 | 0.349 |
| EGFR mutation | Exon 19 vs exon 21 vs others | 0.797 | 0.344 |
| TP53 alterations | Missense variant vs others | 0.675 | 0.506 |
| EXON 4/7 | Yes vs no | 0.37 | 0.748 |
| EXON 8 | Yes vs no | 0.837 | 0.334 |
| Concomitant other mutations | Yes vs no | 0.741 | 0.423 |
